# Salvage Strategy for Long-Term Central Venous Catheter-Associated *Staphylococcus aureus* Infections in Children

**DOI:** 10.3389/fped.2018.00427

**Published:** 2019-01-25

**Authors:** Fanny Alby-Laurent, Cécile Lambe, Agnès Ferroni, Nadège Salvi, David Lebeaux, Morgane Le Gouëz, Martin Castelle, Florence Moulin, Xavier Nassif, Olivier Lortholary, Martin Chalumeau, Julie Toubiana

**Affiliations:** ^1^Department of General Pediatrics and Infectious Diseases, Necker-Enfants Malades University Hospital, AP-HP, Paris Descartes University, Sorbonne Paris Cité, Paris, France; ^2^Department of Pediatric Gastroenterology, Hepatology and Nutrition, Necker-Enfants Malades University Hospital, AP-HP, Paris Descartes University, Sorbonne Paris Cité, Paris, France; ^3^Department of Clinical Microbiology, Necker-Enfants Malades University Hospital, AP-HP, Paris Descartes University, Sorbonne Paris Cité, Paris, France; ^4^Department of Pediatric Critical Care and Anesthesia, Necker-Enfants Malades University Hospital, AP-HP, Paris, France; ^5^Necker-Pasteur Center for Infectious Diseases and Tropical Medicine, Necker-Enfants Malades University Hospital, AP-HP, Paris Descartes University, Sorbonne Paris Cité, Paris, France; ^6^Pediatric Hematology-Immunology Unit, Necker-Enfants Malades University Hospital, AP-HP, Paris Descartes University, Sorbonne Paris Cité, Paris, France; ^7^Department of Pediatric Intensive Care Unit, Necker-Enfants Malades Hospital, AP-HP, Paris, France

**Keywords:** bacteremia, central venous catheters, catheter-related infection/microbiology, child, *Staphylococcus aureus*

## Abstract

**Introduction:** Current international guidelines strongly recommend catheter removal in case of *S. aureus* central line-associated bloodstream infection (CLASBI), but a catheter salvage strategy may be considered in children given age-related specificities. No data is available regarding the outcome of this strategy in children. This study aims to evaluate catheter salvage strategy in children with *S. aureus* CLABSI, and to determine treatment failure rates and associated risk factors.

**Methods:** We retrospectively analyzed data for all children <18 years having *S. aureus* CLABSI on a long-term central venous catheter in a tertiary hospital from 2010 to 2014. We defined catheter salvage strategy as a central venous catheter left in place ≥3 days after initiation of empiric treatment for suspected bacteremia, and catheter salvage strategy failure as the persistence or relapse of bacteremia with a *S. aureus* strain harboring the same antibiotic susceptibility pattern, or the occurrence or the worsening of local or systemic infectious complication between 72 h and 28 days after the first positive blood culture.

**Results:** During the study period, 49 cases of *S. aureus* CLABSI on long-term central venous catheters were observed in 41 children (including 59% with long-term parenteral nutrition) and 6 (15%) isolates were resistant to methicillin. A catheter salvage strategy was chosen in 37/49 (76%) cases and failed in 12/37 (32%) cases. Initial presence of bloodstream co-infection, serum concentration of vancomycin under the targeted value and inadequate empiric treatment were significantly associated with catheter salvage therapy failure.

**Conclusions:** The catheter salvage strategy of *S. aureus* CLABSI on a long-term central venous catheter was frequent in the studied hospital and failed only in one third of cases.

## Introduction

Long-term central venous catheters (CVC) are essential for the management of many chronic diseases requiring prolonged vascular access. The use of long-term CVC is associated with serious complications such as central-line associated/related bloodstream infection (CLABSI/CRBSI) (1). The incidence of CLABSI in children varies from 0.2 to 11 per 1,000 catheter-days (2), depending on the device type, the medical background of the patient, and the clinical setting. *Staphylococcus aureus*, the second most common infectious agent after coagulase-negative staphylococci, is responsible for 10 to 25% of CLABSI (3).

CLABSI caused by *S. aureus* is frequently associated with treatment failure (~50%) and complicated by suppurative thrombophlebitis, endocarditis or metastatic infection (up to 40%) (4–7). Therefore, the Infectious Diseases Society of America (IDSA) guidelines strongly recommend catheter removal in case of *S. aureus* CRBSI or CLABSI in adults, and treating with intravenous (iv) adequate antibiotic therapy for at least 14 days (8). Pediatricians are however often tempted to avoid catheter removal because of the inconvenience of catheter replacement in children, such as risks of general anesthesia, difficulties of vascular access, and the risk of relapse of a CLABSI on the new catheter (9). Thus, IDSA guidelines leave the possibility to keep the CVC in pediatric cases of catheter-related infection if there are “unusual extenuating circumstances” by using combined systemic antibiotics and antibiotic lock therapy (8).

Data regarding the frequency and success rate of the catheter salvage strategy (CSS) for *S. aureus* CLABSI in children with long-term CVC are scarce, and predictors of failure of such a strategy have not been investigated yet. Such a lack of data precludes any clear practice guidelines and adequate selection of children in whom CSS may have the highest chances of success. Here, we report a study on the epidemiology of CLABSI caused by *S. aureus* in children with long-term CVC hospitalized in a tertiary center in France, with a specific analysis of CSS and its predictors of failure.

## Materials and Methods

### General Methodology

We conducted a retrospective observational study in a tertiary care teaching hospital between January 2010 and December 2014. The study was approved by the local ethics committee (ref. 2017-JT-16) and by the French supervisory authority [Comité National de l'Informatique et des Libertés (CNIL); ref. 13685]. We used the Strengthening the Reporting of Observational studies in Epidemiology (STROBE) guidelines to report results (see Table S1). We collected clinical and laboratory data for all children <18 years who had long-term CVC (i.e., tunneled catheter, implantable venous access device or tunneled dialysis catheter) and with a proven or probable *S. aureus* CLABSI. The identification of potential participants was performed by retrospectively recovering all positive blood cultures for *S. aureus* using the microbiological laboratory informatics system and electronic medical files. Data for demographic, clinical characteristics and treatments were directly extracted from patient medical records by one author (FA).

### Definitions

Proven CLABSI was defined according to IDSA criteria as catheter-related bloodstream infection (CRBSI) (8), i.e., bacteremia in a patient who has an intravascular device and ≥1 positive result of culture of blood samples obtained from the peripheral vein, clinical manifestations of infection, and no other apparent source for bloodstream infection, associated with one of the following: a positive result of quantitative catheter segment culture, whereby the same organism was isolated from the catheter segment and the peripheral blood sample; or differential time to positivity between blood cultures obtained from the catheter and peripheral blood. Probable CLABSI was defined according to CDC criteria (10) i.e., bacteremia in a patient who has an intravascular device used within the preceding 48 h, and ≥1 positive result of culture of blood samples (obtained from catheter and/or peripheral vein), clinical manifestations of infection and no apparent other source for bloodstream infection.

*S. aureus* was identified using ApiStaph 20NE strips (BioMerieux, Marcy-l'Etoile, France) or MALDI-TOF mass spectrometry (Andromas system, Paris, France). *In vitro* antibiotic susceptibility testing was assessed as recommended by the European Committee on Antimicrobial Susceptibility Testing (EUCAST, http://www.eucast.org/) (11). Infections were considered hospital-acquired when they occurred after 48 h of hospitalization.

Antibiotic treatment was considered adequate if it included at least one iv antibiotic agent to which the isolate was susceptible according to EUCAST (11) and under the recommended dose (12). The antibiotic lock therapy procedure followed our local recommendations, i.e., the catheter lumen was filled with a volume of antibiotic calculated according to the type of CVC. The final concentration of antibiotic solutions reached 1 to 5 mg/mL (8), and the dwell time needed to reach at least 12 h for at least three consecutive days, to be considered adequate. Only adequate lock therapies were reported.

CSS was defined as long-term CVC left in place at least 3 days after initiation of empiric antibiotic therapy for suspected bacteremia (13).

Failure of CSS was defined as either (i) persistence of positive blood culture(s), (ii) persistence of clinical manifestations of infection without any other apparent source for infection than the catheter (7), (iii) occurrence or worsening of local or systemic infectious complication undetected at the time of the diagnosis, or (iv) relapse defined by a positive blood culture with a *S. aureus* strain harboring the same antibiotic susceptibility pattern within the 4 weeks after the first negative blood culture (8). Local infectious complications included abscess or tunnel infection from the catheter exit site, along the subcutaneous tract of a tunneled catheter (8). Systemic infectious complications included severe sepsis or septic shock (14), suppurative thrombophlebitis, endocarditis, and metastatic foci of infection (8).

Catheter repair was sometimes performed according to the “glued” procedure (15). Target serum concentrations of vancomycin were considered efficient if they were ≥15 mg/L before the 72nd h of treatment (16).

### Statistical Analyses

Chi-square and Mann-Whitney tests were used to compare the distributions of patients' and infections' characteristics between successful and failed CSS, using SPSS v21 (SPSS Inc., Chicago, IL).

## Results

### Patients' Characteristics

**Figure 1:**
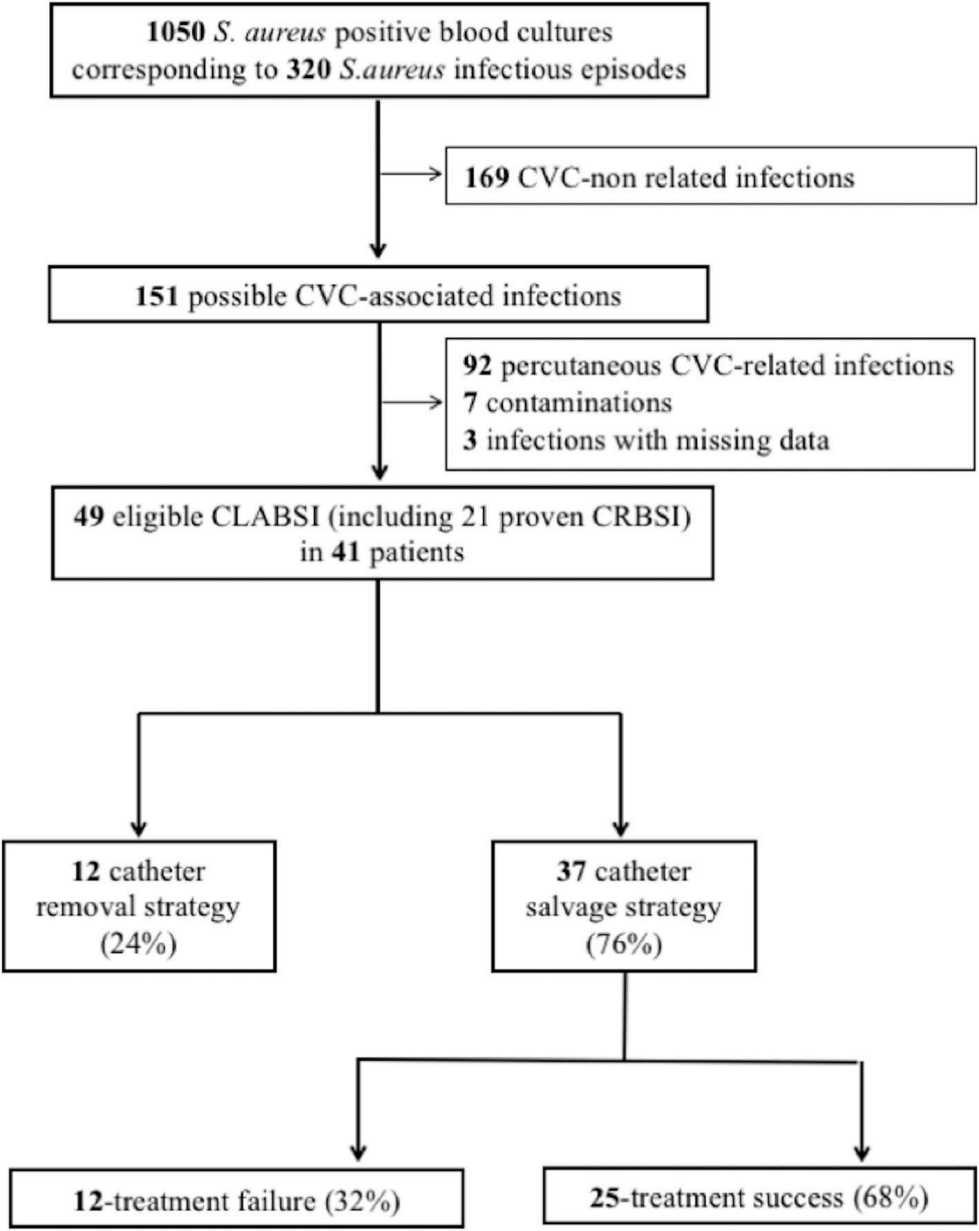
Flow chart from patient identification to data analyzed. CLABSI, central-line associated bloodstream infections; CRBSI, catheter-related bloodstream infections; CVC, central venous catheter.

During the study period, 1,050 blood cultures were positive for *S. aureus*, corresponding to 320 distinct infectious episodes. Forty-nine of these infections occurred in patients carrying long-term CVC and met the criteria of *S. aureus* CLABSI; 21 of them were proven (CRBSI) and 28 were probable CLABSI (Figure 1). Forty-one patients displayed 49 episodes of CLABSI, which includes 6 patients with two, and one patient with three distinct episodes (i.e., > 28 days between the 2 episodes, and all but one patient on a distinct CVC). Sex ratio (M/F) was 0.7, and 24 patients (59%) had a gastrointestinal pathology requiring parenteral nutrition, 14 (34%) an immune deficiency, six (15%) neurologic disorders, and six (15%) a congenital skin disease. The main indication for long-term CVC was prolonged parenteral nutrition (66%).

Median age at the time of infection was 1.4 years [range, 0.1–16.9] (Table S2). CLABSI occurred on tunneled catheters in 80% of cases, on implantable venous access devices in 14% of cases and on tunneled dialysis catheters in 6% of cases, after a median length of use of 22 days [range, 0–548], and 35 (71%) of them were hospital-acquired infections. At the time of CLABSI diagnosis, 15 (31%) children had uncomplicated local manifestations (erythema or purulent hole without tunnel infection), 8 (16%) severe sepsis, and 3 (6%) septic thrombosis. Blood cultures from the CVC were positive in 46/49, and peripheral blood cultures in 27/31 infectious episodes. Culture of the catheter segment was positive in 10 out of the 24 cultured catheters. Methicillin-resistant *S. aureus* (MRSA) strains were isolated in 6 cases (12%) and resistance was not statistically associated with hospital-acquired infections, nor with previous antibiotic therapy. *S. aureus* was found in association with other pathogens in 9 CLABSI (18%) [coagulase-negative *Staphylococci* (*n* = 4), *Enterococcus faecalis* (*n* = 3), Enterobacteriaceae (*n* = 2), *Moraxella catarrhalis* (*n* = 1), and *Pseudomonas aeruginosa* (*n* = 1)]. Initial antibiotic treatment was considered inadequate in 2 cases.

### Catheter Salvage Strategy

Among the 49 CLABSI, CVC was removed in 12 (24%) cases within the first 72 h (Figure 1), one of them was complicated with osteomyelitis under appropriate antibiotics 3 days after CVC removal. Implantable venous access devices were significantly more frequently removed within the first 72 h (*p* = 0.03). CVC was maintained in 37 episodes (76%). Among them, 33 (89%) were treated empirically with vancomycin, 3 (8%) with daptomycin monotherapy, one with cloxacillin (Table 1). Cloxacillin or vancomycin was combined with an aminoglycoside or rifampicin in 14 cases (38%), and with cephalosporins, carbapenems, or daptomycin in 11 (30%) patients. Among the 33 episodes treated with empiric vancomycin, 79% had continuous infusion through the infected CVC, and serum concentrations reached targeted values in 60% of 25 assays.

**Table 1:** Characteristics of the 37 *S. aureus* CLABSI treated with a catheter salvage strategy and risk factors for failure.

**N (%) otherwise stated**	**All *n* = 37**	**Failure *n* = 12**	**Success *n* = 25**	P^*^
**GENERAL CHARACTERISTICS**				
Age, year; median [range]	1.6 [0.2–16.9]	1.5 [0.3–6.9]	1.6 [0.2–16.9]	0.85
Male	14 (38)	5 (42)	9 (36)	0.74
Immunosuppression	11 (30)	4 (33)	7 (28)	0.74
Tunneled catheter	32 (86)	10 (83)	22 (88)	0.70
Days of catheter use; median [range]	22 [0–568]	30 [0–548]	15 [0–468]	0.25
Previous episode(s) of CLABSI	19 (51)	8 (67)	11 (44)	0.20
**INFECTIOUS EPISODE**				
Hospital-acquired infection	26 (70)	8 (67)	18 (72)	0.74
Local symptoms at onset	11 (30)	4 (33)	7 (28)	0.74
Severe sepsis at onset	6 (16)	4 (33)	2 (8)	0.05
CRP at onset (mg/L); median [range]	62 [6–245]	57 [7–165]	73 [6–254]	0.76
CRP at 48–72 h (mg/L)^#^; median [range]	18 [6–93]	7 [6–49]	19 [6–93]	0.26
Polymicrobial infection	8 (22)	5 (42)	3 (12)	0.04
Positive blood cultures from CVC; median [range]	2 [0–6]	2 [0–6]	2 (1–3)	0.49
Positive peripheral blood cultures; median [range]	1 [0–3]	1 [0–3]	0 [0–2]	0.41
*S. aureus* methicillin resistant strain	6 (16)	2 (17)	4 (16)	0.96
**EMPIRIC TREATMENT**				
Cloxacillin	1 (3)	0 (0)	1 (4)	0.48
Vancomycin	33 (89)	9 (75)	24 (96)	0.05
Adapted vancomycin serum concentration^&^	15 (60)	2 (29)	13 (72)	0.04
Inadequate empiric treatment	2 (5)	2 (17)	0 (0)	0.04
Combined therapy	14 (38)	4 (33)	10 (40)	0.70
Antibiotic locks	4 (11)	2 (17)	2 (8)	0.43
Repair kit	5 (14)	3 (25)	2 (8)	0.16

* P-value of the Chi-2 or Mann-Whitney test between success and failure;

# 9 missing data;

& *8 missing data; anti-staphylococcal antibiotic combined with rifampicin or aminoglycosides; CLABSI, central-line associated bloodstream infections*.

Antibiotic locks (glycopeptides or aminoglycosides, *n* = 4) or kit repairs (*n* = 5) were associated with antibiotic therapy in 9 cases (Table 1). Antibiotic susceptibility testing was performed for all infectious episodes. Empiric therapy was considered adequate in 35 cases (95%), and inadequate in two [daptomycin monotherapy, minimal inhibitory concentration (MIC) considered resistant according to EUCAST, http://www.eucast.org/] (11). Seventy-two hours after treatment initiation, all patients received adequate antibiotic therapy according to the antibiotic susceptibility testing, with cloxacillin in 22 infections (59%), vancomycin in 12 (33%), daptomycin in 2 (5%), and 3rd-generation cephalosporin in one case (3%). Median duration of intravenous antibiotic therapy was 15 days [range, 10–45 days]. Three patients had relay oral therapy (cotrimoxazole, rifampicin + ciprofloxacin or clindamycin) for an additional median duration of 15 days [range, 3–19 days]. Nineteen CVCs were removed after 3 days of treatment but within the 28-day period of follow up.

Failure of CSS was observed in 12 (32% of cases) infectious episodes (Table 1). Failure was due to persistence of positive blood culture in 5 patients, septic thrombosis in 2 patients (worsening and new-onset thrombosis), relapse of bacteremia in 3 patients, endocarditis in one patient (right-sided endocarditis without septic metastasis, diagnosed at day 6 of the first positive blood culture), tunnel infection in one patient, and isolated persistent fever without any other apparent source of infection in one patient. CVC was removed for all of them but 3. No mortality attributable to *S. aureus* CLABSI was observed.

### Predictors of CSS Failure

General characteristics and underlying comorbid conditions were not statistically different between patients with failed or successful CSS (Table 1). CLABSI characteristics significantly associated with CSS failure were polymicrobial infection at the time of diagnosis (42 vs. 12%, *p* = 0.04), serum concentration of vancomycin under the targeted value (71 vs. 28%, *p* = 0.04), and inadequate empiric treatment (17 vs. 0%, *p* = 0.04). Severe sepsis at infection onset (33 vs. 8%, *p* = 0.05) tended to be associated with CSS failure.

## Discussion

We report for the first time that CSS for long-term CVC-related *S. aureus* infections was frequent (76%) in a French university hospital with a success rate for CSS of 68%. The potential benefits from CSS include less vascular damage by new catheterization, less exposure to general anesthesia, and less painful procedures. These results contrast with IDSA guidelines that strongly recommend catheter removal for *S. aureus* CLABSI but mention the possibility to maintain the catheter in children “when facing extenuating unusual circumstances such as difficulties of vascular access” (8). Previous reports from cohorts of immune-deficient adult patients have also found high levels (75–86%) of non-compliance to IDSA removal guidelines (13, 17, 18). The only two available pediatric studies reported 64% (19) and 73% (20) of CSS among 112 and 77 infectious episodes, respectively; however, these studies mixed both short-term and long-term CVC-related infections (19). CSS seems to be used more often than suggested by IDSA, which is driven by the high risks of local and general complications of *S. aureus* CLABSI compared to CLABSI caused by other bacterial agents (4, 5, 21). Thus, CSS must prove to be safe and effective.

In our study, the CSS failure rate was 32%. This rate can be considered high compared to those (<10%) observed in non-*S. aureus* bacterial CLABSI (22, 23) but lower than those reported in *S. aureus* CLABSI in adults (40–50%) (4, 13, 18, 19, 21) or one pediatric study (44%) (19). Discrepancies in failure rates may be related to different definitions of CSS failure (20), the lower incidence of MRSA and the frequent use of continuous vancomycin administration through the CVC (79%) observed in our study. This modality of administration provides *in situ* antibiotic concentration required for an optimal antibiotic lock (8, 24). Antibiotic lock has a theoretical effect on CLABSI (25, 26) but is technically difficult to process on a single-lumen long-term CVC (2, 27). The main cause of CSS failure was persistent positive blood culture (14%) and relapse (8%), a finding consistent with previous studies in adult patients (4, 7, 13, 17, 18). Catheter-associated thrombosis, a common complication of *S. aureus* CLABSI that can reach a rate of 70% (28) and is often complicated by secondary hematogenous metastatic infections and subsequent morbidity (6, 27), was here less frequent (6%) but might have been underestimated, as ultrasonography was not systematically performed in routine. Indeed, it is well known that physical examination has a low sensitivity for the diagnosis of thrombosis (29). In our study, no death was attributable to *S. aureus* CLABSI, which differs from the unique pediatric study that reported a crude mortality of 11% (19). This discrepancy can be partly explained by differences in patients' clinical background, as there were no premature infants and a higher rate of children with gastrointestinal disease in our study. One severe complication was observed in the CSS group: endocarditis. The risk of such severe complication should be taken into consideration while evaluating the benefit of a 68% success rate of CSS, although CSS might not be the direct cause of endocarditis it should be investigated for any *S. aureus* CLABSI with systematic early ultrasonic cardiography. Accurate prediction of failure with validated risk factors could help in guiding CSS in CLABSI caused by *S. aureus* in children with long-term CVC.

Several factors associated with an increased risk for CSS failure were identified. Among them, polymicrobial bacteremia was reported to be associated with more severe illness than *S. aureus* bacteremia alone (29, 30). Severe sepsis at onset, which did not reach statistical significance in our study, was found to be a factor of treatment failure in previous studies, and current guidelines recommend CVC removal in such cases (5, 8). Adequate treatment, in particular adapted vancomycin serum concentration, is known to be crucial for treatment success (3). Finally, there is growing evidence of non-susceptibility to daptomycin within *S. aureus* strains, in particular in patients repeatedly exposed to vancomycin therapy (31) such as patients with long-term CVC. Though the size of our study was small, our findings suggest considering daptomycin only in specific cases of vancomycin-intermediate *S. aureus* and after daptomycin susceptibility testing. MRSA was not found as a risk factor of failure, but our MRSA prevalence is much lower than previous findings (16 vs. 36%) (5, 13).

Our retrospective single center study had several limitations. The retrospective design of our study was associated with missing data. As the study was conducted in a single tertiary care center, it is underpowered and the between-centers variability of clinical practices is not evaluable. Thus, confirmation of our findings on patients from different centers is required. In addition, only 40% of the included infections were proven CRBSI that are used as baseline definitions in current guidelines (8, 22, 23). This could be explained by the high rate of CSS, and the reluctance to obtain peripheral blood samples from children. However, limiting our study to CRBSI would have led to additional bias given the low-test performance of differential time to positivity for the diagnosis of *S. aureus* CRBSI (32).

In conclusion, CSS of *S. aureus* CLABSI on a long-term CVC was frequent in the studied pediatric hospital, succeeded in two thirds of cases and was associated with some clinical and treatment characteristics. These results may balance the IDSA guidelines that strongly recommend catheter removal for *S. aureus* CLABSI. The single serious complication observed calls for very close clinical, biological and ultrasonography monitoring of any decision of CSS. Further studies are needed to confirm risk factors of CSS failure that may help to guide decision-making, and the potential beneficial role of antibiotic continuous infusion with a loading dose and a close monitoring of serum concentrations if antibiotic locks cannot be performed.

## Author Contributions

JT and MCh: supervised the work. JT, DL, OL, and MCh: designed the study and the methodology. CL, AF, NS, ML, MCa, FM, XN: gave data from their patients. FA-L: collected data. JT and FA-L: analyzed data. FA-L, JT, and MCh: drafted the manuscript. All authors reviewed and approved the final version of the manuscript.

### Conflict of Interest Statement

The authors declare that the research was conducted in the absence of any commercial or financial relationships that could be construed as a potential conflict of interest.
