# Supplementary material for: Salvage Strategy for Long-Term Central Venous Catheter-Associated Staphylococcus aureus Infections in Children
Source: Front Pediatr. 2019 Jan 25;6:427. doi: 10.3389/fped.2018.00427 (PMC6355702; doi:10.3389/fped.2018.00427)
Supplement: Supplementary file 2 [file Table_2.DOCX]

**Supplementary Table S2. Characteristics of infections at the time of diagnosis**

| N (%) otherwise stated | | **All cases**  **n=49** |  |
| --- | --- | --- | --- |
| **Age at the time of infection, yr; median** [**range**] | | 1.4 [0.1-16.9] | |
| **Type of CVC** | |  | |
|  | Tunneled ± sleeved | 39 (80) | |
|  | Implantable venous access device | 7 (14) | |
|  | Dialysis catheter | 3 (6) | |
| **Days of catheter use; median** [**range**]^*^ | | 22 [0-548] | |
| **Hospitalized-acquired infection** | | 35 (71) | |
| **Department of hospitalization** | |  | |
|  | Gastroenterology | 18 (37) | |
|  | Intensive care units | 10 (20) | |
|  | Immunology/hematology | 9 (18) | |
|  | Other^¶^ | 12 (25) | |
| **Clinical manifestations at onset** | |  | |
|  | Local infection | 15 (31) | |
|  | Severe sepsis | 8 (16) | |
|  | Deep vein thrombosis | 3 (6) | |
| ***S. aureus* susceptibility^§^** | |  | |
|  | Methicillin | 43 (88) | |
|  | Rifampicin | 49 (100) | |
|  | Ciprofloxacin | 43 (88) | |
|  | Cotrimoxazole | 48 (98) | |
|  | Aminoglycosides | 45 (92) | |
| **Polymicrobial infection** | | 9 (18) | |
| **Empiric treatment** | |  | |
|  | Vancomycin | 42 (86) | |
|  | Cloxacillin | 3 (6) | |
|  | Daptomycin monotherapy | 4 (8) | |
|  | Combined therapy with rifampicin or aminoglycosides | 19 (40) | |
|  | Inadequate empiric treatment^*^ | 2 (4) | |
| **Catheter removal** | | 12 (24) | |
| *1 missing data; ^#^ Long-term parenteral treatment or dialysis;  ^¶^ General pediatrics, nephrology, metabolic disease and surgery departments; ^§^ Susceptible on antibiotic susceptibility testing according to EUCAST (11) | | | |
